# Adhesion of Composite Resin to Additively Manufactured Zirconia Using a Self-Adhesive Resin Cement: Evaluation of Micro-Shear Bond Strength

**DOI:** 10.3390/polym18182305

**Published:** 2026-09-20

**Authors:** Yue Zhu, Chao Qian

**Affiliations:** 1 Department of Prosthodontics, Shanghai Ninth People’s Hospital, Shanghai Jiao Tong University School of Medicine, Shanghai 200011, China; zyyy99@sjtu.edu.cn; 2 College of Stomatology, Shanghai Jiao Tong University, Shanghai 200011, China; 3 National Center for Stomatology, Shanghai 200011, China; 4 National Clinical Research Center for Oral Diseases, Shanghai 200011, China; 5 Shanghai Key Laboratory of Stomatology, Shanghai 200011, China; 6 Shanghai Research Institute of Stomatology, Shanghai 200011, China; 7 Department of Stomatology, Shanghai Putuo Central Hospital, Shanghai 200062, China

**Keywords:** zirconia, resin, additive-manufactured, shear bond strength

## Abstract

Effective bonding between zirconia ceramic restorations and resin remains a clinical challenge, particularly in the context of intraoral repair and cementation. This study aimed to investigate the micro-shear bond strength (μSBS) of stereolithography (SLA)- and digital light processing (DLP)-fabricated zirconia–resin cement-composite resin bonding specimens. Rectangular zirconia specimens (10 × 5 × 5 mm^3^) were fabricated by SLA, DLP, and computer numerical control (CNC) milling technologies. The surface roughness (Ra) of zirconia was measured after air abrasion. A composite resin cylinder (5 mm in height and 1 mm in diameter) was added to each zirconia substrate for the μSBS test. The failure mode images were obtained using a scanning electron microscope. The adhesive, cohesive, and mixed failure patterns were analyzed. The Ra and μSBS values were statistically analyzed with one-way ANOVA followed by S-N-K post hoc comparisons (a = 0.05). The Ra of SLA (1.05 ± 0.03 μm) and DLP (1.02 ± 0.03 μm) zirconia after air-abrasion were significantly higher than that of CNC (0.91 ± 0.02 μm) (*p* < 0.001). The SLA (23.37 ± 4.66 MPa) and DLP (23.24 ± 3.7 MPa) groups exhibited similar μSBS values for the bonding specimens to those of the CNC group (21.13 ± 3.48 MPa) (*p* = 0.237), indicating that the bond strength of the zirconia–resin cement-composite resin assembly for SLA and DLP zirconia was comparable to that for CNC zirconia.

## 1. Introduction

Computer numerical control (CNC) milling has long been the mainstream method for fabricating zirconia ceramics, offering technical maturity and production efficiency. However, as a subtractive process, it involves raw material wastage, tool wear, microcracking, and geometric constraints [1]. Additive manufacturing (AM) has recently emerged as an alternative digital fabrication technology. Through a layer-by-layer deposition process, AM technology enables high material efficiency and the fabrication of geometrically complex structures unattainable by CNC milling [2]. Among all zirconia-based materials, yttria-stabilized tetragonal zirconia polycrystal (Y-TZP) was widely used in prosthetic dentistry due to the high durability and favorable mechanical properties [3]. Preliminary research has been conducted on the fabrication of zirconia restorations using 3D printing technology. Several studies have employed techniques such as stereolithography (SLA) and digital light processing (DLP) to manufacture zirconia restorations from ceramic suspensions [4]. The results demonstrated that the mechanical properties of these 3D-printed restorations were comparable to those produced by computer numerical control (CNC) milling and could meet clinical requirements [5,6]. It has been reported that SLA and DLP techniques could produce zirconia restorations that meet the clinical standards of dimensional accuracy and marginal fit [7].

Due to the chemical inertness and the absence of a glass phase, conventional ceramic bonding strategies, such as acid etching and silane application, were ineffective in facilitating adhesion to the surface of zirconia ceramic [8]. Researchers have found that zirconia restorations exhibit inferior bonding performance and are more prone to debonding than glass-ceramic restorations [9]. Effective bonding of resin to zirconia requires surface roughening to provide micromechanical interlock, along with sufficient wettability and chemical affinity to achieve chemical coupling [10]. The functional monomer 10-methacryloyloxydecyl dihydrogen phosphate enables stable chemical bonds of the adhesive to zirconia through the phosphate ester group [11]. The fabrication process plays a critical role in determining the surface properties. Studies have demonstrated that the zirconia restorations fabricated using an SLA- or DLP-based technique showed significantly greater surface roughness compared to the milled ones [7]. In addition, SLA employs a point-by-point laser scanning mode, while DLP uses an area light source to polymerize an entire layer at once [12,13]. The point exposure mode and laser-induced slurry evaporation in SLA increase the void formation within each layer. These voids in the green body may persist after sintering, manifesting as surface pores and microcracks that alter the surface topography [14]. Consequently, it is reasonable to hypothesize that differences in manufacturing methods may lead to different bonding behaviors. However, a systematic theoretical framework regarding the resin bonding capacity of printed zirconia has yet to be fully developed. To achieve durability of bonding for zirconia restoration, adhesive resin, and dental substrates, it is essential to first establish a clear understanding of the surface characteristics and the immediate resin bonding performance of zirconia.

The bond strength of adhesive systems is typically evaluated using the shear bond strength (SBS) test [15]. Compared with traditional SBS experiments, the micro-shear bond strength (μSBS) test reduces non-uniform stress distribution and enables the detection of subtle differences in adhesive performance [16]. Although the results of the μSBS test may not fully reflect clinical conditions due to the simplification in geometry and loading configuration, it still serves as a widely used and efficient screening test for bond strength evaluation [17].

**Scheme 1:**
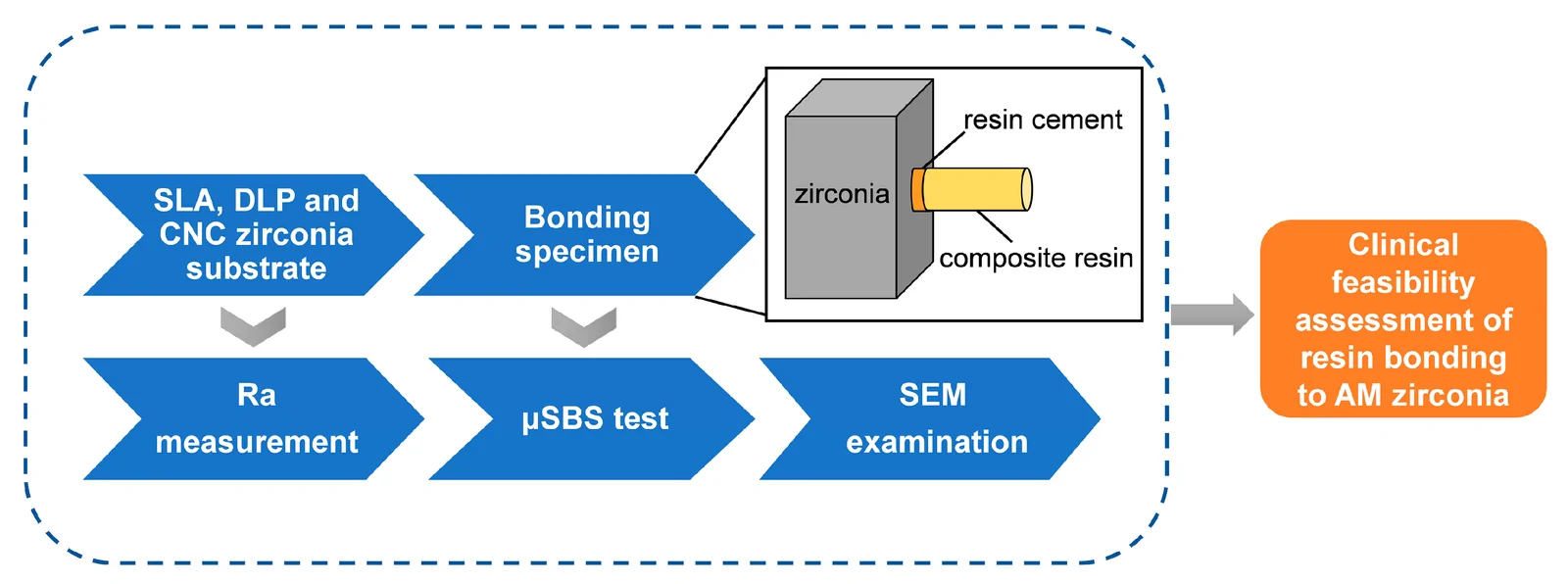
Schematic summary of the study design and significance.

Therefore, the aim of this research was to investigate the bond strength of zirconia–resin cement-composite resin assembly for SLA, DLP, and CNC zirconia. The null hypothesis of this study posited that no significant difference would exist in the μSBS between the SLA, DLP, and CNC groups. The research framework and significance of this study are depicted in Scheme 1.

## 2. Materials and Methods

### 2.1. Preparation of Zirconia Specimens

**Figure 1:**
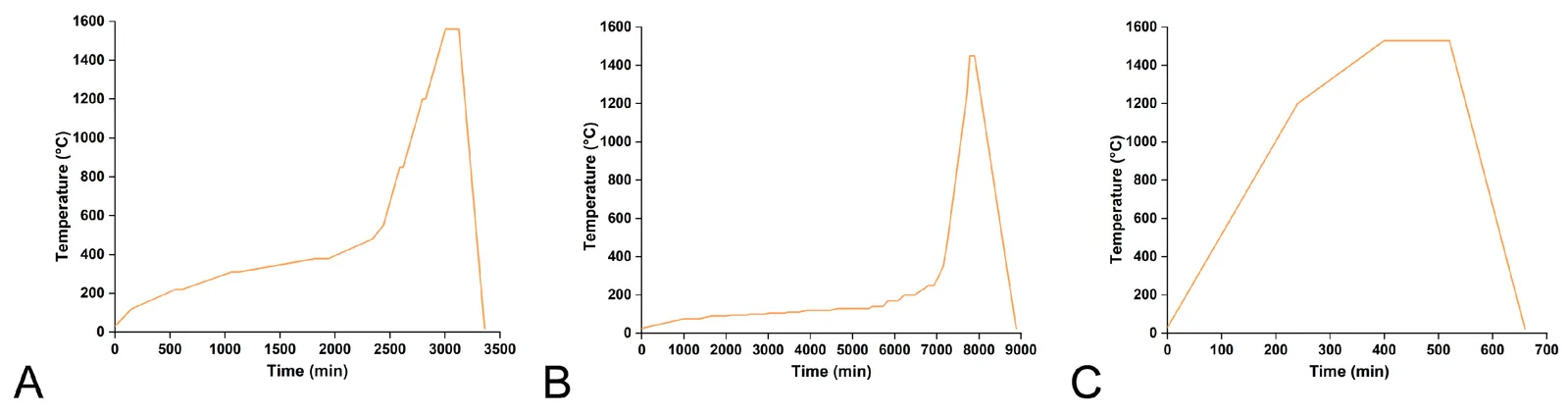
Sintering schedule for SLA (**A**), DLP (**B**), and CNC (**C**) zirconia.

Fifteen rectangular zirconia specimens (10 × 5 × 5 mm^3^) were fabricated using SLA, DLP, and CNC technologies, comprising 5 specimens per material. The manufacturing procedures of zirconia specimens were the same as those used in our earlier study [18]. Information on the printable zirconia suspension and the zirconia block is listed in Table 1. Both the SLA zirconia slurry (BLM-FTC-1, PORIMY, Kunshan, China) and the DLP zirconia slurry (LithaCon 3Y 230 D, Lithoz, Vienna, Austria) had a solid concentration of over 50 vol%. The 3D model of the specimen was designed based on the shrinkage and saved as a standard tessellation language (STL) file. For printed zirconia, an SLA (MultPRTer SL100, PORIMY, Kunshan, China) and a DLP (Cerafab 7500, Lithoz, Vienna, Austria) system were used to photopolymerize zirconia suspensions with a layer thickness of 25 μm. The detailed manufacturing parameters are shown in Table 2. All green bodies were removed from the support structures and sintered, with SLA sintering at the highest temperature of 1500 °C for 2 h and DLP at 1450 °C for 2 h. For CNC zirconia, zirconia blocks were milled using a CAD/CAM dental milling system (DWX-52DCi, Roland DG, Hamamatsu, Japan) and sintered at the highest temperature of 1530 °C for 2 h. The complete sintering schedules of the three materials are shown in Figure 1. The surfaces with dimensions of 10 × 5 mm^2^ of the zirconia specimens were ground and polished following the sequence of P600, P800, and P1000 SiC abrasive papers under water, with 30 s for each grit. All specimens were ultrasonically cleaned in distilled water at 40 kHz for 10 min. The polished surfaces of each specimen were subjected to air-abrasion using 50 μm aluminium oxide particles (Cobra, Renfert GmbH, Hilzingen, Germany) at 2 bar pressure for 15 s using a fine blasting unit (Gobi Comfort-2, Wassermann Dental-Maschinen GmbH, Hamburg, Germany). The air-abrasion nozzle was kept perpendicular to the zirconia surface at a distance of 10 mm. A dental triple syringe was used to clear the residual particles.

### 2.2. Surface Roughness Measurement

One treated surface of each specimen was randomly selected for surface roughness (Ra) measurement by a contact profilometer (Perthometer M1, Mahr GmbH, Göttingen, Germany). The cut-off length was 0.8 mm and the evaluation length was 4 mm. Five measurements at different regions were recorded for each specimen and the mean Ra value was calculated. The Ra value for each group was calculated as the average of the values obtained from the five specimens.

### 2.3. Preparation of Zirconia–Resin Cement-Composite Resin Bonding Specimens

Fifteen composite resin cylinders (1 mm in diameter and 5 mm in height) were prepared for each group by a customized 3D-printed resin mold. The mold had holes with an inner diameter of 1 mm and a depth of 5 mm. A thin layer of alginate separating agent (3DS, Shanghai New Century Dental Materials Co., Ltd., Shanghai, China) was applied to the inner walls of the holes. After the separating agent was dried, the flowable composite resin Filtek™ Z350 XT (shade A2, 3M ESPE, St. Paul, MN, USA), a methacrylate-based nanocomposite, was condensed into the hole. The resin specimens were light-cured using a light-emitting diode (LED) device (Elipar, 3M ESPE, St. Paul, MN, USA) with an irradiance of 1000 mW/cm^2^ on both the top and bottom sides for 5 s before being demolded. Then they were subjected to an additional 30 s of light curing from all directions. All specimens were placed at room temperature for 24 h to ensure complete polymerization.

Three resin cylinders were bonded to each zirconia specimen, with one cylinder attached to each of the three rectangular surfaces. To bond the zirconia and composite resin, RelyX™ U200 (shade A2, 3M ESPE, St. Paul, MN, USA), a dual-cure self-adhesive methacrylate-based resin cement containing functionalized phosphoric acid monomers, was mixed and applied to one side of the resin cylinder. The composite resin specimen was positioned at the center of the zirconia surface under uniform pressure with a finger. The excess resin cement was removed using a micro-brush. The light curing was done for 40 s under the same light curing conditions. All zirconia–resin bonding specimens were stored at room temperature for 24 h before testing.

### 2.4. Micro-Shear Bond Strength Test

**Figure 2:**
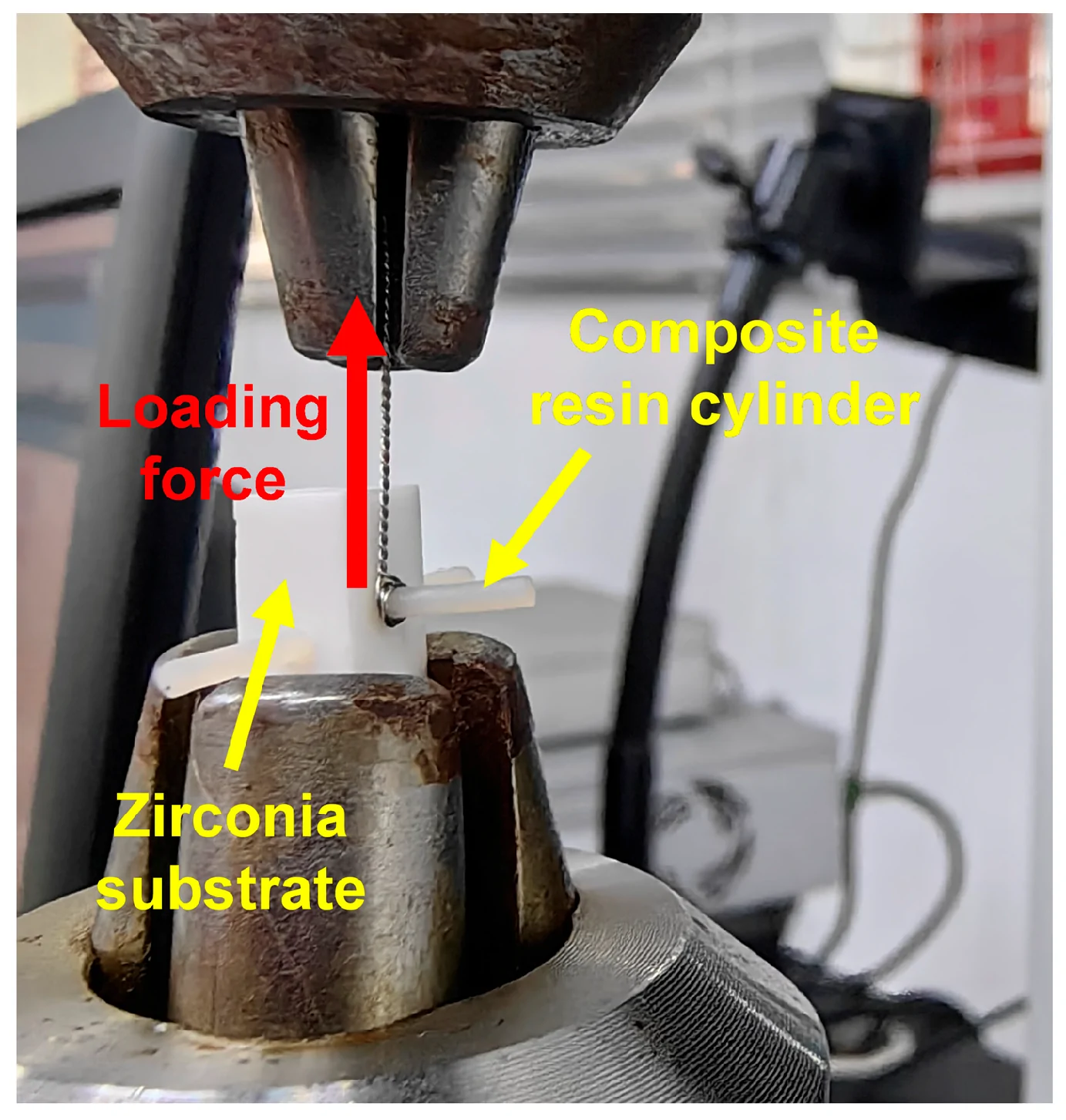
Bonding specimen subjected to micro-shear bond strength test using wire-loop loading.

The micro-shear bond strength test was conducted employing the wire and loop method [15]. The μSBS test device is shown in Figure 2. A 0.2 mm diameter orthodontic ligature wire was tied to each resin cylinder. One side of the wire needed to be as close as possible to the resin-zirconia interface. The other side was fixed to the piston of a universal test machine (5ST, Tinius Olsen, Horsham, PA, USA). The interface, wire, and piston were connected in alignment to ensure proper load distribution. The shear load was applied at a crosshead speed of 0.5 mm/min until failure. The μSBS (MPa) was calculated by dividing the maximum fracture load (N) by the bonding area (0.785 mm^2^).

### 2.5. Failure Mode Analysis

A scanning electron microscope (SEM) (MFP-3D Origin, Asylum Research, Santa Barbara, CA, USA) was used to observe the specimen surfaces after polishing, air abrasion, and the μSBS test. For the fractured surfaces, the failure was determined to be in three modes: cohesive, where fractures occurred exclusively within the resin/cement; adhesive, where fractures occurred at the interface between resin/cement and zirconia; and mixed, where both adhesive and cohesive failure occurred [19].

### 2.6. Statistical Analysis

The statistical analyses were performed by IBM SPSS Statistics 26 software at a level of significance of a = 0.05. The Shapiro–Wilk test and Levene test were utilized to confirm the normality and homogeneity of variance of μSBS and Ra data respectively. One-way analysis of variance (ANOVA) was carried out to compare the μSBS and Ra values between three groups, followed by S-N-K post hoc comparisons. The frequency of failure modes between groups was compared by a chi-square test.

## 3. Results

### 3.1. Surface Properties

Table 3 lists the means and standard deviations of the Ra values after air-abrasion. According to the results of the one-way ANOVA, the material significantly influenced the surface roughness (*p* < 0.001). The CNC zirconia had the lowest Ra value (0.91 ± 0.02 μm) among the three materials. The SLA (1.05 ± 0.03 μm) and DLP (1.02 ± 0.03 μm) zirconia had similar and higher Ra values.

**Figure 3:**
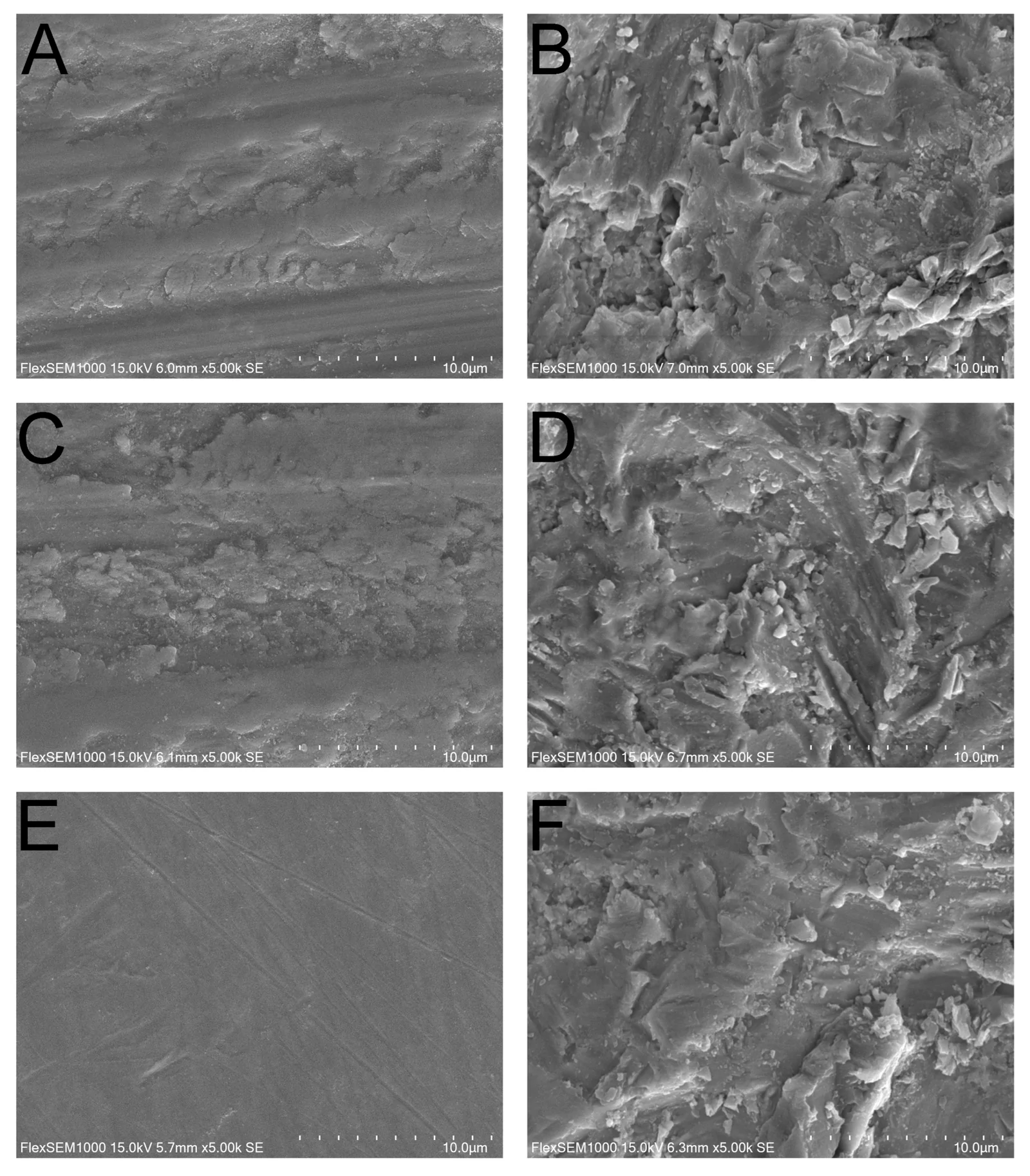
Scanning electron microscope (SEM) images of zirconia substrates after surface treatments at a magnification of 5000×. SLA (**A**), DLP (**C**), and CNC (**E**) zirconia after polishing; SLA (**B**), DLP (**D**), and CNC (**F**) zirconia after air-abrasion.

The SEM results of zirconia surfaces before and after air-abrasion are shown in Figure 3. Without air-abrasion, both SLA and DLP zirconia exhibited uneven surfaces composed of blocky or granular structures even after polishing. The CNC zirconia showed a smooth milled surface with faint scratches. After air-abrasion, the surface images of the three materials all presented a coarse texture covered with large, irregular sandblasting particles.

### 3.2. Micro-Shear Bond Strength

The means and standard deviations of the μSBS values are presented in Table 4. The μSBS value of SLA was 23.37 ± 4.66 MPa, the value of DLP was 23.24 ± 3.75 MPa, and the value of CNC was 21.13 ± 3.48 MPa. One-way ANOVA showed that there was no significant difference between the μSBS values of the SLA, DLP, and CNC groups (*p* = 0.237).

**Figure 4:**
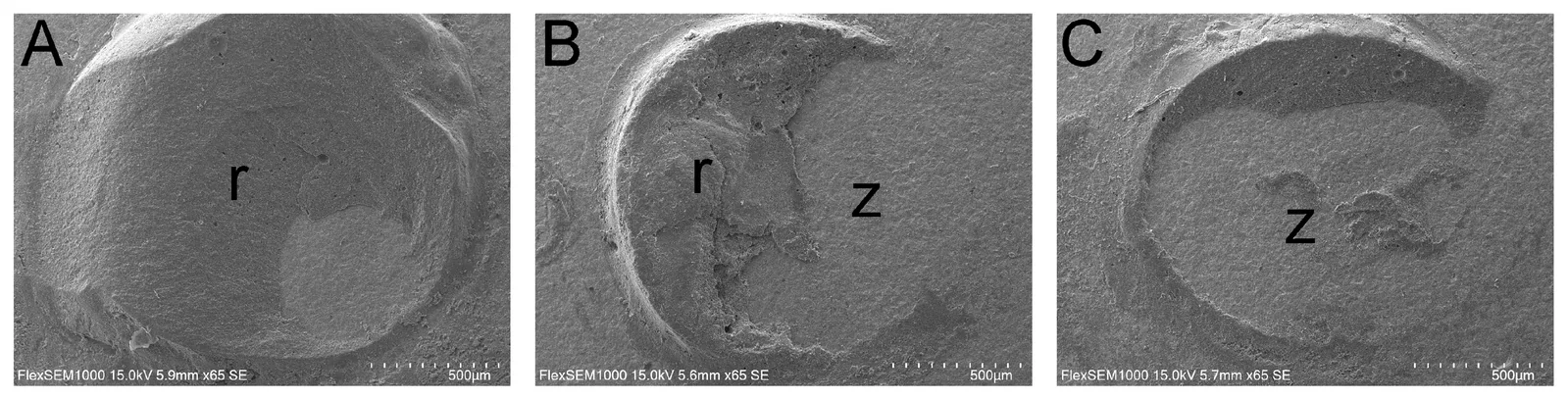
SEM images of cohesive failure (**A**), mixed failure (**B**), and adhesive failure (**C**) at a magnification of 65×. r, resin; z, zirconia.

Figure 4 shows representative SEM images of the three failure modes. In cohesive failure, the fracture was observed in the resin/cement, with more than 80% of the resin remaining on the zirconia surface. In adhesive failure, most resin/cement was detached, with less than 20% left, exposing most of the zirconia surface. The mixed failure combined both fractured resin/cement and exposed substrate.

**Figure 5:**
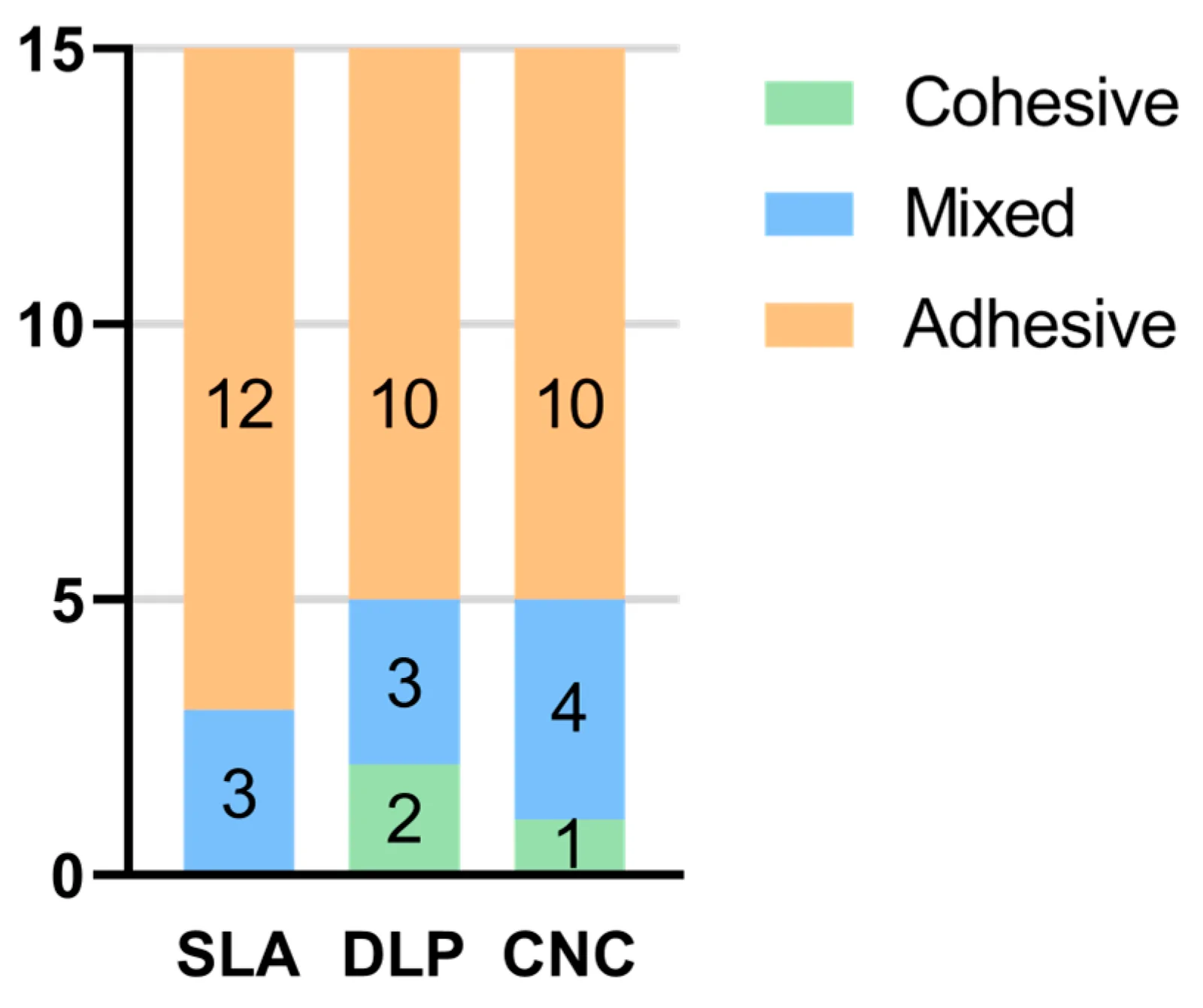
Frequency of failure modes in each group after the µSBS test. The numbers in each bar are the counts of specimens in each failure mode.

As shown in Figure 5, the predominant failure mode was adhesive failure at the zirconia–resin/cement interface, accounting for 80% in SLA, 66% in DLP, and 66% in CNC. DLP exhibited the highest count of cohesive failure at 13%, followed by CNC at 6% and SLA at 0%. For mixed failure, CNC had a count of 26% while SLA and DLP each constituted 20%. However, no significant difference was found in the counts of failure modes among SLA, DLP, and CNC zirconia according to the chi-square test (χ^2^ = 2.45, *p* = 0.654).

## 4. Discussion

The present study demonstrated that the μSBS values among the SLA, DLP, and CNC groups did not differ significantly. As a result, the null hypothesis could not be rejected. The micro-shear bond strength test was considered an effective method for evaluating the interfacial bonding efficacy of dental restorative materials [17]. The μSBS values for the SLA and DLP groups were comparable and both exceeded those of the CNC group. Despite this trend, no statistically significant differences were detected among the three groups, implying a slight advantage for additively manufactured zirconia over milled zirconia. Therefore, the additive manufacturing approach did not adversely affect the bonding performance of zirconia to resin. Consistent with the study by Kessler et al. [20], no significant interactions were observed in the bond strength between manufacturing methods and ceramic materials under various bonding conditions. Furthermore, both bond strength values and surface properties remained unaffected by manufacturing technique and material type.

SEM images showed that the SLA and DLP zirconia exhibited uneven surfaces composed of blocky or granular structures even after the same polishing procedures as CNC zirconia. After air abrasion, all zirconia specimens exhibited surfaces covered with coarse blasting particles, with no visible differences, which might account for the comparable μSBS values observed among the three groups. However, the topographical similarity alone is insufficient to fully explain the bonding behavior. Additional measurements of contact angle and surface free energy would be valuable to further elucidate the bonding mechanisms.

The SLA and DLP groups exhibited significantly higher Ra values after air-abrasion (1.05 ± 0.03 µm for SLA and 1.02 ± 0.03 µm for DLP) than CNC zirconia (0.91 ± 0.02 µm), which might be explained by their inherently rougher surfaces. As reported in our earlier work [18], the Ra values of SLA, DLP, and CNC zirconia before air-abrasion were 0.38 ± 0.03 µm, 0.37 ± 0.04 µm, and 0.16 ± 0.00 µm respectively. Though air-abrasion created a dominant roughening effect, it may not entirely erase the effect of the manufacturing method. The initial surface defects and microstructural heterogeneities may remain beneath the blasted layer and influence the final surface properties. Studies have also reported that SLA and DLP zirconia possess rougher surfaces than CNC zirconia [21]. The disparity in surface morphology reflects the intrinsic differences in fabrication techniques. The layer-by-layer curing process in vat polymerization techniques could create multi-level or stair-step surface structures, increasing the surface roughness of zirconia [7,21]. In addition, printing artifacts, such as incomplete curing or residual pixelation, might also contribute to the irregular surface topography [22].

In vitro studies reported that the tetragonal phase of Y-TZP might transform to the monoclinic phase, with volume expansion under the high stresses caused by sandblasting, introducing microcracks and affecting the surface integrity [23]. According to Ravi et al. [24], air-abrasion with particle sizes equal to or less than 100 μm and air pressures lower than 4 bar can induce protective compressive stresses, which counteract the damage induced by air-abrasion. The pressure of 2 bar has been identified as the optimal pressure for achieving reliable and durable resin-zirconia bonding [25]. The present study employed a pressure of 2 bar for SLA, DLP, and CNC zirconia, resulting in comparable μSBS values across all groups. However, the precise influence of air-abrasion on the surface microstructure and phase stability of SLA and DLP zirconia remains unclear. Future work should include phase analysis to directly verify whether abrasion-induced monoclinic phase transformation occurs and to what extent it correlates with the bonding performance.

The observed failure modes revealed cohesive failure, mixed failure, and adhesive failure. Among these, cohesive failure is often recognized as an indicator of strong adhesion among zirconia, resin cement, and composite resin [26]. In the bonding specimens, both the CNC group and the DLP group exhibited all three failure modes, whereas the SLA group demonstrated only mixed and adhesive failure modes. Statistical analysis showed no significant differences in the frequency of failure modes among the three groups. The adhesive failure was the predominant mode across all three groups. SEM images revealed that the exposed zirconia substrate remained intact during adhesive failure, which aligns with the generally weak bonding performance of zirconia [27]. Enhancing surface treatment and modifying zirconia surface topography could improve interfacial bondability and facilitate the failure mode shift from adhesive to cohesive, potentially lowering the clinical debonding risk [28,29,30].

The fundamental nature of bonding strength mainly depends on micromechanical interlocking between resin cement and zirconia. Air abrasion is an effective and practical clinical method to enhance surface roughness, surface energy, and wettability. However, it may induce microcracks and phase transformations, compromising mechanical strength [31]. There is no clear consensus on the optimal method for improving the bond strength of AM zirconia. Recent studies have investigated the application of surface texturing, which has been shown to significantly enhance resin–zirconia bonding [29]. According to Liu et al. [32], zirconia surfaces exhibited a notable increase in both immediate and aging SBS when engineered with various textures via nanoparticle jetting technology, such as circular, square, and triangular patterns. DLP technology has also been proven to successfully print groove and hexagon grids on zirconia surfaces to improve the bond strength [33,34]. It is confirmed that these microstructures expand the bonding area beyond air-abrasion-induced microroughness [29]. Compared to other traditional methods to promote micromechanical retention, additive manufacturing provided a one-step and non-destructive process to prepare a desirable surface texture [34].

Several limitations of this in vitro study should be acknowledged. A wider variety of zirconia-cement-composite adhesive systems should be investigated to help determine whether the observed bonding performance of SLA and DLP zirconia is universally applicable. This study evaluated the immediate bond strength between SLA and DLP zirconia and resin, with no aging treatment applied to the specimens. In the oral environment, the composite resin is continuously exposed to water absorption, saliva contamination, and masticatory loading, which can promote hydrolytic degradation and weaken the ceramic–resin interface [16]. Further studies involving aging protocols or intraoral simulation are warranted to assess the durability of the bonding in SLA and DLP zirconia–resin cement-composite resin systems. Also, the standardized specimen lacks the complexity of anatomical crowns, which may affect the stress distributions. This highlights the necessity of investigating crown-shaped specimens. Moreover, future research should aim to leverage the advantages of additive manufacturing by correlating printed surface topography with resin bonding performance, thereby ensuring durable and reliable adhesion for AM zirconia restorations.

## 5. Conclusions

Within the limitations of this study, the following conclusions can be drawn:

1. The shear bond strength of SLA- and DLP-fabricated zirconia–resin cement-composite resin assembly was comparable to that of the CNC zirconia counterpart.
2. The predominance of adhesive failure indicated that the bonding interface remained the weak point in the SLA and DLP zirconia–resin cement-composite resin assembly, highlighting the need for further optimization of the surface treatment or fabrication process.

## Figures and Tables

**Table 1:** Manufacturers and components of SLA, DLP, and CNC zirconia.

Material	Name, Manufacturer	Main Components (wt%)			
		ZrO_2_ + HfO_2_ + Y_2_O_3_	Y_2_O_3_	Al_2_O_3_	Other Oxides
SLA	BLM-FTC-1, PORIMY	98.39	6.05	0.241	1.195
DLP	LithaCon 3Y 230 D, Lithoz	≥99.0	>4.5 to ≤6.0	≤0.5	≤0.5
CNC	ST, UPCERA (Shenzhen, China)	>99.0	4.5–6.0	<0.5	<0.5

**Table 2:** Manufacturing parameters of SLA, DLP, and CNC zirconia.

Material	SLA	DLP	CNC
Manufacturing device	MultPRTer SL100	Cerafab 7500	DWX-52DCi
Enlargement ratio	1.266	1.283	1.250
Build orientation	Horizonal	Horizonal	-
Layer thickness	25 μm	25 μm	-
Light source	UV laser	LED	-
Wavelength	355 nm	385 nm	-
Scanning speed	>300 layers/h	1.6 s/layer	-
Resolution	40 μm	40 μm	-

**Table 3:** Surface roughness (Ra) values of three groups with ANOVA results.

Group	*n*	Ra (μm) Mean ± SD	F	*p*
SLA	15	1.05 ± 0.03 ^a^	33.296	<0.001
DLP	15	1.02 ± 0.03 ^a^		
CNC	15	0.91 ± 0.02 ^b^		

Different superscript letters (a, b) in the column indicate significant differences among the three groups (S-N-K test, *p* < 0.05). Values sharing the same superscript letter (a) have no significant difference.

**Table 4:** SBS values of the three groups with ANOVA results.

Group	*n*	μSBS (MPa) Mean ± SD	F	*p*
SLA	15	23.37 ± 4.66	1.488	0.237
DLP	15	23.24 ± 3.75		
CNC	15	21.13 ± 3.48		

## Data Availability

The raw data supporting the conclusions of this article will be made available by the authors upon request.

## Abbreviations

The following abbreviations are used in this manuscript:

μSBS	Micro-shear bond strength
SLA	Stereolithography
DLP	Digital light processing
CNC	Computer numerical control
AM	Additive manufacturing
Y-TZP	Yttria-stabilized tetragonal zirconia polycrystal
STL	Standard tessellation language
SEM	Scanning electron microscope
ANOVA	One-way analysis of variance
